# Lower admission reverse shock index multiplied by Glasgow coma scale score is associated with early hematoma expansion in spontaneous intracerebral hemorrhage

**DOI:** 10.3389/fneur.2026.1947152

**Published:** 2026-09-01

**Authors:** Shukai Wu, Bingji Chen, Jiayin Wang

**Affiliations:** Department of Neurosurgery, Second Affiliated Hospital of Fujian Medical University, Quanzhou, China

**Keywords:** Glasgow coma scale, hematoma expansion, prediction, reverse shock index, risk stratification, spontaneous intracerebral hemorrhage

## Abstract

**Background:**

Early hematoma expansion (HE) contributes to neurological deterioration after spontaneous intracerebral hemorrhage (sICH), but simple bedside tools for early risk stratification remain limited. This study examined the association between the reverse shock index multiplied by Glasgow Coma Scale score (rSIG) and early HE and evaluated its predictive performance.

**Methods:**

Consecutive adults with sICH admitted to a single center between July 2016 and June 2026 were retrospectively included. rSIG was calculated as Glasgow Coma Scale score × systolic blood pressure/heart rate using first admission measurements. HE was defined as an absolute hematoma-volume increase >6 mL or a relative increase >33% on follow-up non-contrast computed tomography within 8 h. Associations were assessed using hierarchical logistic regression, restricted cubic splines, quartile analysis, bootstrap resampling, and 1:2 matched sensitivity analysis.

**Results:**

Of 2,450 patients screened, 2,035 were included, and 156 (7.7%) developed early HE. Each 5-point decrease in rSIG was independently associated with higher odds of HE in the fully adjusted model (adjusted odds ratio, 1.783; 95% confidence interval, 1.560–2.038; *p* < 0.001). The association was nonlinear and most pronounced at low rSIG values (P for nonlinearity <0.001). The rSIG-only model yielded an area under the curve of 0.828 (95% confidence interval, 0.789–0.864). The optimal cutoff was 17.44, with 78.2% sensitivity and 80.2% specificity. Calibration was acceptable, and decision curve analysis indicated net benefit across threshold probabilities of approximately 1.7–30%. Findings remained consistent in bootstrap and matched analyses.

**Conclusion:**

Lower admission rSIG was independently and nonlinearly associated with early HE after sICH. rSIG may provide a rapid bedside aid for early risk stratification and complement imaging assessment, although prospective.

## Introduction

Spontaneous intracerebral hemorrhage (sICH) is one of the most devastating forms of stroke and continues to impose a substantial burden of mortality and long-term disability worldwide, despite advances in acute stroke and neurocritical care (1, 2). A major determinant of early clinical deterioration is hematoma expansion (HE), a dynamic process that occurs predominantly during the first several hours after symptom onset and affects approximately one fifth of patients within 24 h (3, 4). HE aggravates intracranial mass effect and secondary brain injury and has been consistently associated with neurological deterioration, mortality, and long-term functional dependence (4, 5). Because the opportunity to limit ongoing bleeding is time-sensitive, prompt identification of patients at high risk of HE is essential for intensified neurological monitoring, timely repeat imaging, correction of coagulopathy, blood pressure management, and selection for targeted management strategies or clinical trials (2). Therefore, a simple and readily available approach to early HE risk stratification remains an important clinical need in the acute management of sICH.

Against this clinical background, substantial efforts have been devoted to identifying patients at risk of HE using neuroimaging markers and multivariable prediction models. The computed tomography (CT) angiography spot sign and non-contrast computed tomography (NCCT) features, including the blend sign, black hole sign, island sign, and intrahaematomal hypodensities, have been associated with subsequent hematoma growth (6–8). However, contrast-enhanced imaging is not feasible for every patient or available in every institution, while individual NCCT markers show variable sensitivity and specificity across study populations (7, 8). Clinical–imaging scores have therefore been developed by integrating imaging findings with variables such as time to initial CT, neurological status, hematoma characteristics, and coagulation parameters (7). Representative tools, including the 9-point HE score and the BRAIN prediction algorithm, combine several clinical and CT-derived variables and have demonstrated useful risk stratification; however, their application requires formal image assessment and completion of multiple predictor inputs (9, 10). More recently, More recently, machine-learning and radiomics-based approaches have demonstrated promising discriminatory performance by extracting high-dimensional information from routinely acquired CT images (11, 12). Nevertheless, these approaches generally require image segmentation, dedicated software, technical expertise, and model-specific validation, which may limit their immediate deployment in emergency or resource-constrained settings. In contrast, rSIG is calculated from only three routinely recorded admission variables—GCS, SBP, and HR—and may be available at first clinical contact, before detailed imaging-marker interpretation or laboratory results. Its potential additional value is therefore operational: rSIG may provide a rapid first-pass physiological screen to identify patients who require expedited imaging review and intensified neurological monitoring. It is intended to complement, rather than replace, CT-based markers, clinical–imaging scores, or radiomics models.

One potential bedside marker is the reverse shock index multiplied by the Glasgow Coma Scale score (rSIG), calculated as Glasgow Coma Scale (GCS) × systolic blood pressure (SBP) / heart rate (HR). By combining the reverse shock index with the GCS score, rSIG integrates hemodynamic status and neurological impairment into a single measure that can be obtained immediately from routine admission observations without additional imaging, laboratory testing, or specialized software (13, 14). Large trauma registries and external validation studies have shown that lower rSIG is associated with a higher risk of mortality and generally provides better discrimination than the shock index or modified shock index (13–15). Its prognostic value has also been demonstrated in patients with severe trauma accompanied by head injury and in broader traumatic brain injury populations, suggesting that rSIG may be particularly informative when neurological dysfunction and physiological instability coexist (16, 17). A nationwide cohort further showed that rSIG could help identify patients requiring massive transfusion, resuscitative procedures, surgery, or other emergency interventions (18). However, existing evidence has focused predominantly on traumatic injury, and the association between admission rSIG and early HE in patients with sICH remains insufficiently characterized. Evaluating rSIG in this population may therefore provide a practical physiological complement to imaging-based HE risk assessment.

Accordingly, the present study conducted a single-center retrospective cohort study to investigate the association between admission rSIG and early HE in patients with sICH. The research aimed to determine whether lower rSIG was independently associated with an increased risk of HE after adjustment for demographic characteristics, onset-to-admission time, comorbidities, and clinically relevant laboratory and imaging variables. The study also examined the potential nonlinear relationship between rSIG and HE and evaluated the discrimination, calibration, and clinical utility of rSIG as a bedside risk-stratification marker. The robustness of the association was further assessed using hierarchical regression models, quartile analysis, bootstrap resampling, and a matched sensitivity analysis.

## Materials and methods

### Study design and participants

This single-center retrospective cohort research enrolled consecutive adult patients with sICH admitted to the Second Affiliated Hospital of Fujian Medical University between July 1, 2016, and June 30, 2026. All patients were managed according to the institutional diagnostic and treatment protocol. sICH was confirmed by baseline NCCT, and follow-up NCCT was routinely performed within 8 h of baseline imaging to assess early HE. The inclusion criteria were: (1) Age ≥18 years; (2) sICH confirmed by baseline NCCT; (3) Initial diagnosis and treatment of the index sICH at the study institution; (4) Baseline hematoma volume >1 mL; (5) Completion of follow-up NCCT within 8 h after the first imaging; and (6) Availability of the clinical, laboratory, physiological, and imaging data required to calculate rSIG and determine early HE. On the other side, the exclusion criteria were: (1) Secondary sICH caused by trauma, intracranial aneurysm, arteriovenous malformation, brain tumor, cerebral venous thrombosis, hemorrhagic transformation of cerebral infarction, or other identifiable causes; (2) Isolated intraventricular hemorrhage without intraparenchymal involvement; (3) Hematoma evacuation, minimally invasive aspiration, or another intervention affecting hematoma volume before follow-up NCCT; (4) Unavailable or poor-quality CT images; and (5) Missing key variables required for the primary analysis, including variables necessary for calculating rSIG, determining HE, or completing the prespecified multivariable analysis. For patients with repeated admissions, only the first eligible hospitalization was included.

### Data collection

Demographic characteristics, medical history, admission findings, laboratory results, and imaging data were obtained from the electronic medical record and radiology systems. The collected variables included age, sex, smoking, alcohol use, onset-to-admission time, body temperature (BT), Glasgow Coma Scale (GCS) score, HR, SBP, hypertension (HP), diabetes mellitus (DM), cerebral herniation, platelet count (PLT), hemoglobin (Hb), red blood cell (RBC) count, absolute lymphocyte count (ALC), high-density lipoprotein cholesterol (HDL-C), low-density lipoprotein cholesterol (LDL-C), creatinine (Cr), uric acid (UA), alkaline phosphatase (ALP), calcium (Ca), total bilirubin (TBil), aspartate aminotransferase (AST), albumin (Alb), blood glucose (BG), total cholesterol (TC), international normalized ratio (INR), and activated partial thromboplastin time (APTT). Admission physiological variables, including SBP and HR, were defined as the first measurements obtained immediately after hospital arrival and before any initial therapeutic intervention or stabilization. Laboratory variables were defined as the first available measurements obtained before surgery or other major therapeutic interventions. Onset-to-admission time was calculated from symptom onset or last-known-well time to hospital arrival.

### Ethics statement

This single-center retrospective study was conducted at the Second Affiliated Hospital of Fujian Medical University. The study protocol was reviewed and approved by the Ethics Committee of the Second Affiliated Hospital of Fujian Medical University (approval number: KY-2026-004). The study was conducted in accordance with the Declaration of Helsinki. Because this study involved the retrospective analysis of routinely collected clinical data and posed no additional risk to participants, the requirement for written informed consent was waived by the Ethics Committee. All patient data were anonymized before analysis, and patient confidentiality was strictly protected.

### Assessment of rSIG and HE

rSIG was calculated as GCS × SBP/HR using the first GCS, SBP, and HR measurements obtained immediately after hospital arrival and before any initial therapeutic intervention or stabilization. The primary outcome was early HE. Eligible patients had sICH with an initial hematoma volume >1 mL and underwent follow-up noncontrast CT within 8 h after baseline CT. Hematoma volume was estimated using the Tada formula (ABC/2). HE was defined as an absolute volume increase >6 mL or a relative increase >33% from baseline. Two experienced neurosurgeons independently reviewed all CT scans while blinded to clinical data, rSIG values, and each other’s assessments. Disagreements were resolved by consensus.

### Statistical analysis

The distribution of continuous variables was assessed using the Shapiro–Wilk test. Because the continuous variables were not normally distributed, they were summarized as medians with interquartile ranges and compared using the Mann–Whitney U test. Categorical variables were presented as numbers and percentages and compared using the chi-square test or Fisher’s exact test, as appropriate. Univariable logistic regression was used to examine the associations between candidate variables and HE. Because GCS, SBP, and HR are mathematical components of rSIG, they were not included as independent covariates in multivariable models containing rSIG. Variables with *p* < 0.10 in univariable analysis were entered into backward likelihood-ratio logistic regression, with rSIG forced into the model. Adjusted odds ratios (aORs) and 95% confidence intervals were reported. The association of rSIG with HE was expressed per 5-point decrease. Onset-to-admission time was entered as a continuous variable on its original scale, with ORs and aORs reported per 100-min increase. RBC count and blood glucose were modeled per 1 × 10^12^/L and 1 mmol/L increase, respectively. The independent association between rSIG and HE was further examined using four prespecified hierarchical logistic regression models. Model 1 was unadjusted. Model 2 included age, sex, and onset-to-admission time. Model 3 additionally included hypertension and DM. Model 4 further included cerebral herniation, PLT, Hb, BG, INR, and APTT. Model discrimination and overall predictive accuracy were evaluated using the area under the receiver operating characteristic curve (AUC), Brier score, and Akaike information criterion. Potential nonlinear associations between rSIG and HE were evaluated using restricted cubic spline (RCS) analysis with four knots placed at the 5th, 35th, 65th, and 95th percentiles of the rSIG distribution. The median rSIG value was used as the reference. Overall and nonlinear associations were evaluated using likelihood-ratio tests. rSIG was also categorized into quartiles, with the highest quartile serving as the reference category. Linear trends were assessed by assigning the median rSIG value of each quartile and entering this variable continuously into the regression model.

The predictive performance of the rSIG-only model was evaluated using ROC analysis, calibration analysis, and decision curve analysis (DCA). The optimal cutoff was determined by maximizing the Youden index, and the corresponding sensitivity and specificity were reported. Calibration was assessed using a smoothed calibration curve, Brier score, calibration intercept, and calibration slope. DCA was used to quantify the net clinical benefit of the rSIG model across a range of threshold probabilities.

Model interpretation was performed using SHapley Additive exPlanations (SHAP) for the final backward stepwise multivariable logistic regression model, which included rSIG, onset-to-admission time, cerebral herniation, RBC count, and blood glucose. SHAp values were calculated on the log-odds scale. Global feature importance was summarized using mean absolute SHAP values, and the direction and distribution of individual feature contributions were visualized using a SHAP summary plot.

A secondary 1:2 nearest-neighbor matched sensitivity analysis without replacement was performed using a logistic-regression-derived matching score based on age, sex, onset-to-admission time on its original scale, hypertension, diabetes mellitus, cerebral herniation, platelet count, hemoglobin, blood glucose, international normalized ratio, and activated partial thromboplastin time. A caliper width of 0.20 standard deviations of the logit of the matching score was used. Covariate balance was assessed using standardized mean differences, with an absolute value <0.10 indicating acceptable balance. The association between rSIG and HE was subsequently examined using conditional logistic regression (LR) stratified by matched set.

All statistical tests were two-sided, and *p* < 0.05 was considered statistically significant. Analyses were performed using Python 3.14.

## Results

### Baseline characteristics

**Figure 1:**
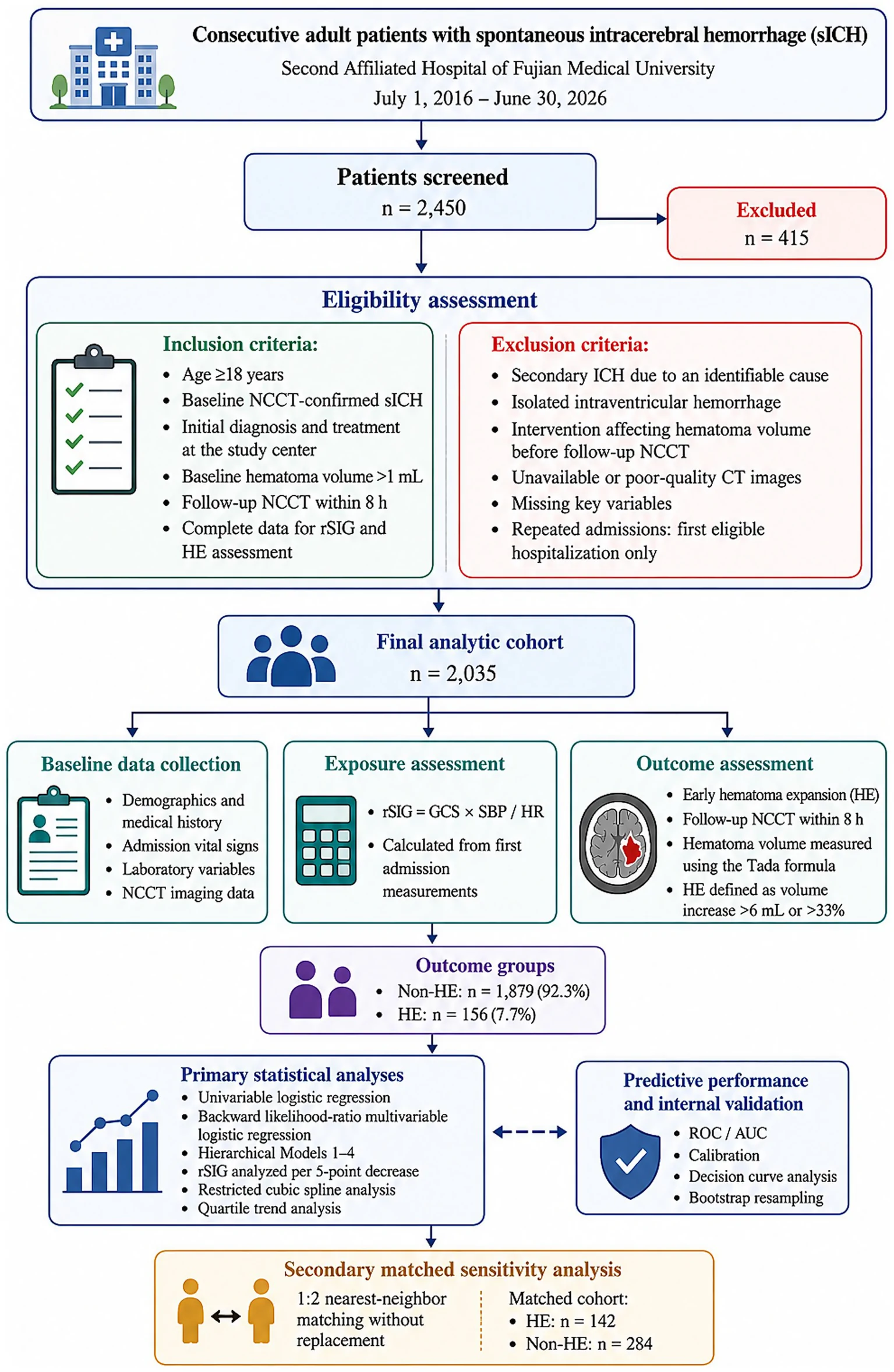
Flow chart.

Of 2,450 patients screened, 415 were excluded and 2,035 were included in the analysis; 156 patients (7.7%) experienced early HE (Figure 1). None of the continuous variables met the normality assumption in both outcome groups and are therefore reported as medians with interquartile ranges (Supplementary Table S2). Compared with patients without HE, patients with HE had a longer onset-to-admission time [96 (10–2,160) vs. 12 (6–48) minutes, *p* < 0.001], lower GCS scores [7.5 (4.0–10.2) vs. 15.0 (13.0–15.0), *p* < 0.001], higher HR [84.5 (78.0–97.0) vs. 80.0 (72.0–88.0) beats/min, *p* < 0.001], and lower rSIG values [11.382 (6.333–16.664) vs. 23.731 (18.854–27.924), p < 0.001]. Cerebral herniation was more frequent in the HE group (19.9% vs. 4.7%, p < 0.001). Several laboratory measures also differed between groups, whereas age, sex, smoking, alcohol use, SBP, HTN, and DM did not (Supplementary Table S1).

### Exploratory multivariable analysis and model interpretation

Because GCS, SBP, and HR are mathematical components of rSIG, so they were excluded from the candidate predictors to avoid structural collinearity. The remaining 27 variables were evaluated using univariable logistic LR. Twenty variables meeting the prespecified screening criterion of *p* < 0.10 were entered into backward likelihood-ratio multivariable LR, with rSIG retained in the model. The final stepwise model included rSIG, onset-to-admission time, cerebral herniation, RBC count, and BG. After mutual adjustment, each 5-point decrease in rSIG was associated with a 74.6% increase in the odds of HE (aOR, 1.746; 95% CI, 1.534–1.988; *p* < 0.001). Longer onset-to-admission time was associated with higher odds of HE (aOR per 100-min increase, 1.053; 95% CI, 1.039–1.067; p < 0.001). Cerebral herniation (aOR, 1.893; 95% CI, 1.098–3.262; *p* = 0.022) and higher BG (aOR per 1 mmol/L increase, 1.076; 95% CI, 1.010–1.146; *p* = 0.024) were also associated with HE, whereas higher RBC count was inversely associated with HE (aOR per 1 × 10^12^/L increase, 0.559; 95% CI, 0.430–0.726; *p* < 0.001). Complete univariable results and the parameter estimates from the final backward stepwise multivariable logistic regression model are presented in Supplementary Table S3. The final model included rSIG, onset-to-admission time, cerebral herniation, RBC count, and blood glucose, and showed good overall performance, with an AUC of 0.864, a Brier score of 0.053, and an AIC of 797.1. To further improve model interpretability, SHAP analysis was performed for this same final stepwise model rather than for a prespecified adjustment model. The SHAP results showed that rSIG was the most influential predictor in the model, making the largest contribution to individual predictions of HE, whereas onset-to-admission time, cerebral herniation, RBC count, and blood glucose provided additional explanatory value (Supplementary Figure S1). In the SHAP summary plot, lower rSIG values were generally associated with a shift toward a higher predicted probability of HE, which was consistent with the multivariable logistic regression results and further supported the robustness of rSIG as an independent risk factor for HE.

### Discrimination, calibration, and clinical utility of rSIG

The predictive performance of rSIG alone was assessed using ROC analysis, calibration analysis, and DCA. The rSIG-only model demonstrated good discrimination, with an AUC of 0.828 (95% CI, 0.789–0.864; *p* < 0.001). The optimal cutoff was rSIG ≤17.44, corresponding to a sensitivity of 78.2%, specificity of 80.2%, and Youden index of 0.584.

**Figure 2:**
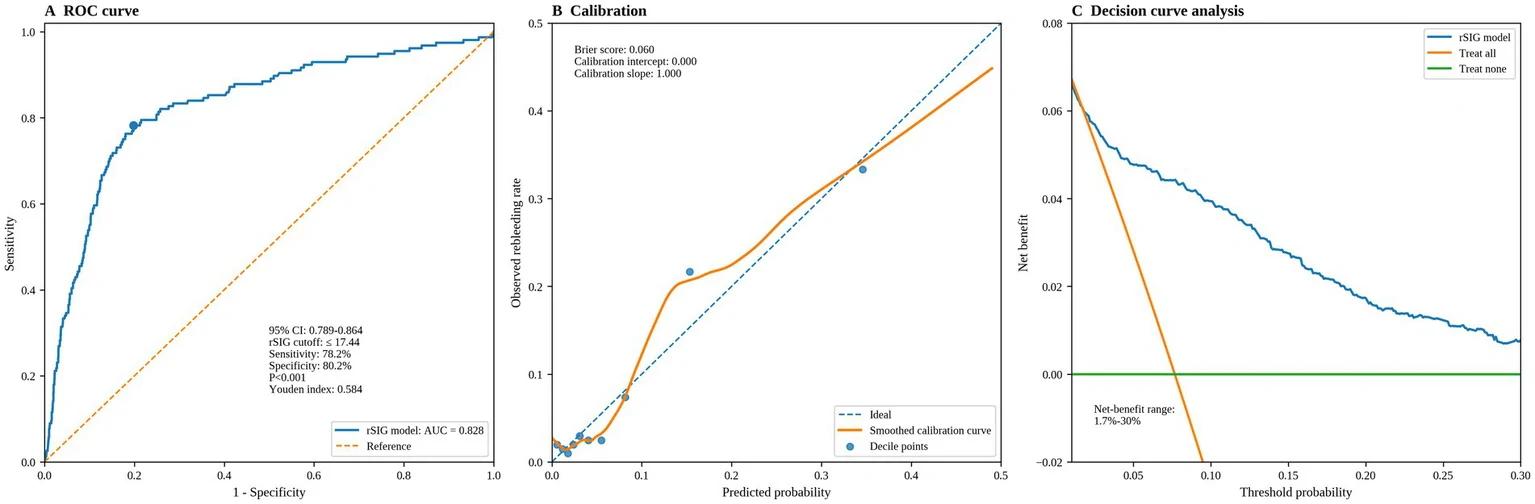
Discrimination, calibration, and clinical utility of rSIG for predicting HE. **(A)** Receiver operating characteristic curve of the rSIG-only logistic regression model. The model achieved an area under the curve of 0.828 (95% CI, 0.789–0.864; *p* < 0.001). The optimal cutoff determined by the maximum Youden index was rSIG ≤17.44, corresponding to a sensitivity of 78.2%, specificity of 80.2%, and Youden index of 0.584. The solid curve represents the rSIG model, the dashed diagonal line represents the reference line, and the point indicates the optimal operating threshold. **(B)** Calibration of the rSIG-only model. The dashed diagonal line represents perfect agreement between predicted and observed probabilities, the solid curve represents the locally smoothed calibration curve, and the points represent observed HE rates across deciles of predicted probability. The model had a Brier score of 0.060, a calibration intercept of 0.000, and a calibration slope of 1.000. **(C)** Decision curve analysis comparing the rSIG model with the treat-all and treat-none strategies. The rSIG model provided greater net benefit than both reference strategies across threshold probabilities of approximately 1.7–30%.

The smoothed calibration curve showed generally close agreement between predicted probabilities and observed HE rates. The model had a Brier score of 0.060, a calibration intercept of 0.000, and a calibration slope of 1.000. DCA indicated that the rSIG model provided greater net benefit than the treat-all and treat-none strategies across threshold probabilities of approximately 1.7–30% (Figure 2).

### Association between rSIG and early HE across hierarchical models

The association between lower rSIG and HE remained consistent across all four prespecified hierarchical logistic regression models. In the unadjusted model, each 5-point decrease in rSIG was associated with more than a twofold increase in the odds of HE (Model 1: OR, 2.212; 95% CI, 1.970–2.483; *p* < 0.001). The association persisted after adjustment for age, sex, and onset-to-admission time (Model 2: aORs, 2.063; 95% CI, 1.830–2.326; *p* < 0.001) and after additional adjustment for hypertension and diabetes mellitus (Model 3: aORs, 2.078; 95% CI, 1.842–2.344; p < 0.001).

**Figure 3:**
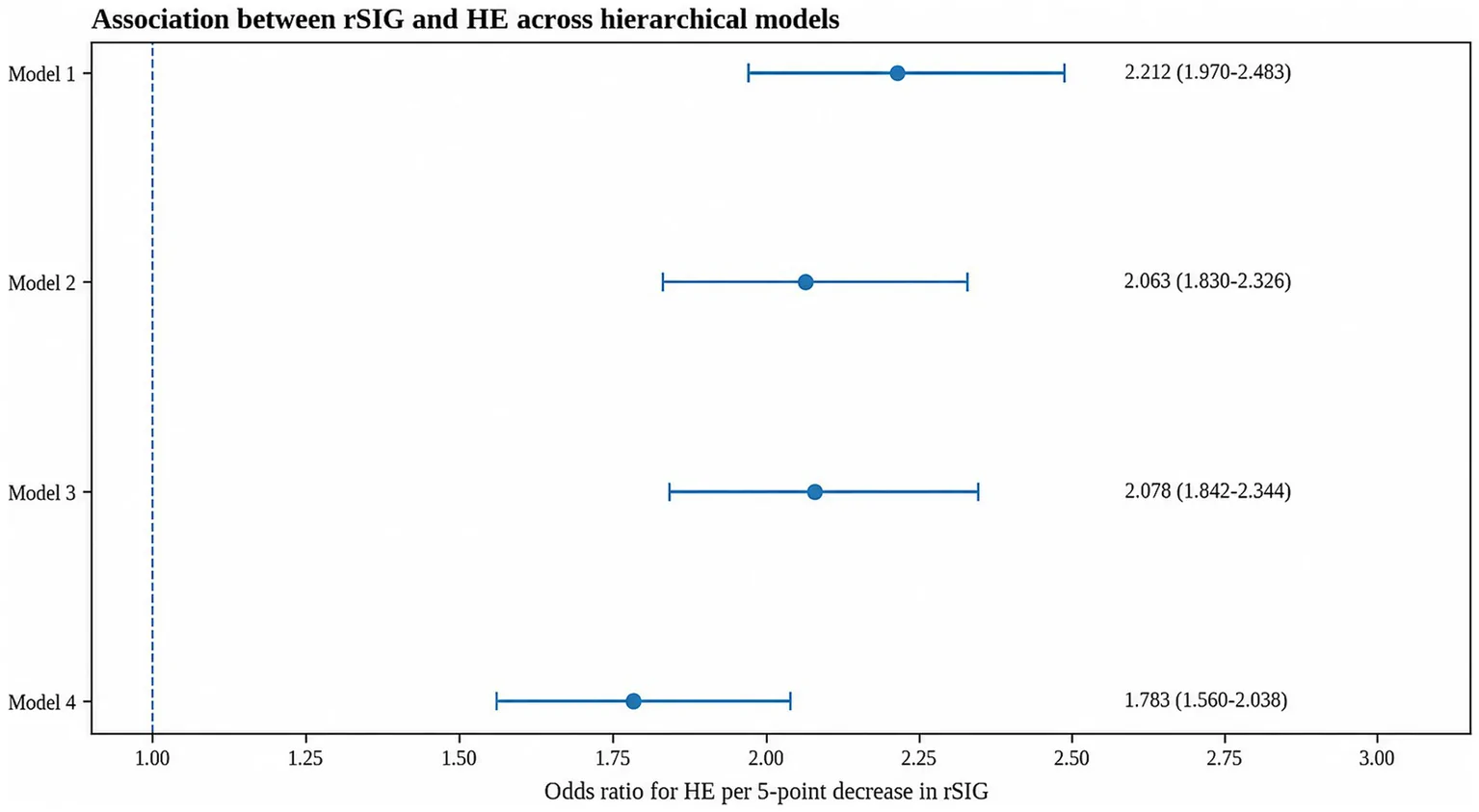
Forest plot of the association between rSIG and HE across prespecified hierarchical logistic regression models. Odds ratios are reported per 5-point decrease in rSIG. The ORs were 2.212 (95% CI, 1.970–2.483) for Model 1, 2.063 (95% CI, 1.830–2.326) for Model 2, 2.078 (95% CI, 1.842–2.344) for Model 3, and 1.783 (95% CI, 1.560–2.038) for Model 4; all *p* values were <0.001. Model 1 was unadjusted. Model 2 adjusted for age, sex, and onset-to-admission time. Model 3 additionally adjusted for hypertension and diabetes mellitus. Model 4 further adjusted for cerebral herniation, PLT, Hb, BG, INR, and APTT. CI, confidence interval; OR, odds ratio; rSIG, reverse shock index multiplied by Glasgow Coma Scale score.

In the fully adjusted model, which further included cerebral herniation, PLT, Hb, BG, INR, and APTT, each 5-point decrease in rSIG remained independently associated with a 78.3% increase in the odds of HE (Model 4: aORs, 1.783; 95% CI, 1.560–2.038; *p* < 0.001). Although progressive covariate adjustment attenuated the magnitude of the association, its direction and statistical significance remained unchanged (Table 1 and Figure 3). Model 4 achieved the highest discrimination, with an AUC of 0.867, a Brier score of 0.053, and an AIC of 809.5.

**Table 1:** Association between rSIG and HE across prespecified hierarchical logistic regression models.

Model	Adjustment	aOR	95% CI	P	AUC	Brier score	AIC
Model 1	Unadjusted	2.212	1.970–2.483	<0.001	0.828	0.060	882.9
Model 2	Age, sex, and onset-to-admission time	2.063	1.830–2.326	<0.001	0.849	0.055	826.9
Model 3	Model 2 plus hypertension and diabetes mellitus	2.078	1.842–2.344	<0.001	0.851	0.055	829.6
Model 4	Model 3 plus cerebral herniation, PLT, Hb, BG, INR, and APTT	1.783	1.560–2.038	<0.001	0.867	0.053	809.5

AIC, Akaike information criterion; APTT, activated partial thromboplastin time; AUC, area under the receiver operating characteristic curve; BG, blood glucose; CI, confidence interval; Hb, hemoglobin; INR, international normalized ratio; OR, odds ratio; PLT, platelet count; rSIG, reverse shock index multiplied by Glasgow Coma Scale score.

### Nonlinear association between rSIG and early HE

RCS analysis demonstrated significant overall and nonlinear associations between rSIG and HE in all four hierarchical models (all P for overall association <0.001; all P for nonlinearity <0.001). Using the median rSIG value of 23.29 as the reference, the odds of HE increased markedly as rSIG decreased.

**Figure 4:**
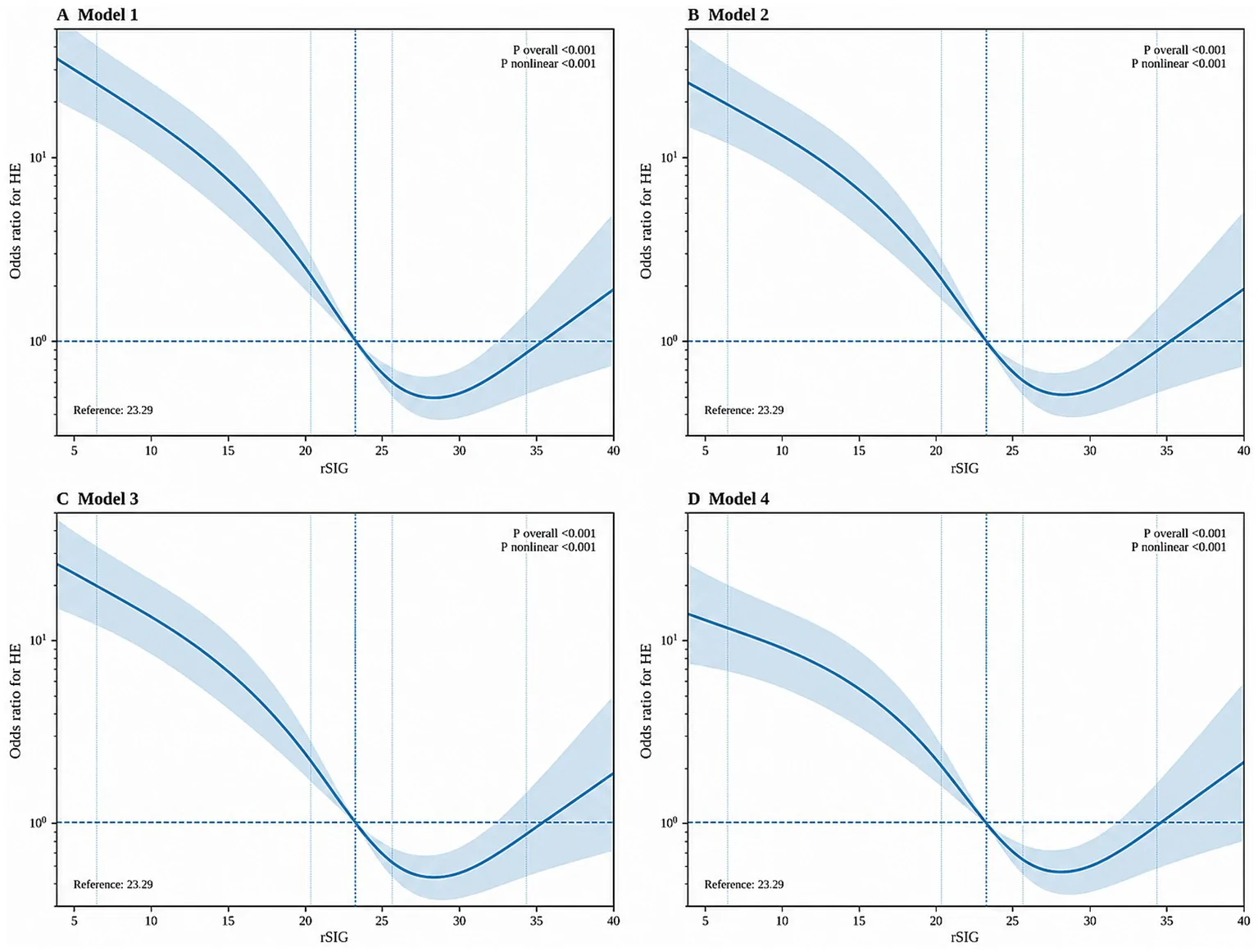
Restricted cubic spline associations between rSIG and HE across four prespecified logistic regression models. **(A–D)** correspond to Models 1–4, respectively. Solid curves represent odds ratios and shaded regions represent 95% confidence intervals. The median rSIG (23.29) was used as the reference value (OR = 1.00). Knots were placed at the 5th, 35th, 65th, and 95th percentiles of rSIG (6.45, 20.36, 25.67, and 34.34, respectively). The overall and nonlinear associations were significant in every model (all *p* overall <0.001; all *p* nonlinear <0.001). In Model 4, the estimated OR was 3.76 (95% CI, 2.47–5.72) at rSIG = 17.44 and 9.06 (95% CI, 5.52–14.89) at rSIG = 10.00. Model 1 was unadjusted. Model 2 adjusted for age, sex, and onset-to-admission time. Model 3 additionally adjusted for hypertension and diabetes mellitus. Model 4 further adjusted for cerebral herniation, PLT, Hb, BG, INR, and APTT. CI, confidence interval; OR, odds ratio; rSIG, reverse shock index multiplied by Glasgow Coma Scale score.

In the fully adjusted model, the estimated OR was 3.76 (95% CI, 2.47–5.72) at rSIG = 17.44 and 9.06 (95% CI, 5.52–14.89) at rSIG = 10.00. Across the lower and middle ranges of rSIG, the spline curves showed a steep decline in HE risk as rSIG increased, followed by a flatter association at higher values. Confidence intervals widened at the upper tail because fewer patients had very high rSIG values; therefore, the apparent increase in risk at the extreme upper range should be interpreted cautiously (Figure 4).

### Sensitivity and robustness analyses

The stability of the stepwise model was evaluated using 1,000 bootstrap resamples. Onset-to-admission time was selected in 100.0% of bootstrap models. RBC, cerebral herniation, and BG were selected in 79.8, 78.7, and 73.8% of models, respectively, with coefficient-direction consistency of at least 99.9%. rSIG was retained in every resampled model because it was forced into the model; therefore, its 100% selection frequency was not interpreted as evidence of variable-selection stability. Nevertheless, its adjusted effect estimate was similar to that obtained from the fully adjusted prespecified model, supporting the robustness of its independent association with HE (Table 2).

**Table 2:** Stepwise regression sensitivity analysis and bootstrap variable-selection stability.

Model	Adjustment variables	OR	95% CI	*p*-value	AUC	Brier score	AIC
Panel A. Association of rSIG across the primary and stepwise sensitivity models							
Model 1	Unadjusted	2.212	1.970–2.483	<0.001	0.828	0.060	882.9
Model 2	Age, sex, and onset-to-admission time	2.063	1.830–2.326	<0.001	0.849	0.055	826.9
Model 3	Model 2 plus hypertension and diabetes mellitus	2.078	1.842–2.344	<0.001	0.851	0.055	829.6
Model 4	Model 3 plus cerebral herniation, PLT, Hb, BG, INR, and APTT	1.783	1.560–2.038	<0.001	0.867	0.053	809.5
Stepwise model	Onset-to-admission time, cerebral herniation, RBC, and BG	1.746	1.534–1.988	<0.001	0.864	0.053	797.1

Variable	Effect scale	Beta	SE	aOR	95% CI	*p*-value
Panel B. Complete results of the final backward stepwise logistic regression model						
rSIG	Per 5-point decrease	0.557	0.066	1.746	1.534–1.988	<0.001
Onset-to-admission time	Per 100-min increase	0.052	0.007	1.053	1.039–1.067	<0.001
Cerebral herniation	Coded 1 vs. 0	0.638	0.278	1.893	1.098–3.262	0.022
RBC	Per 1 × 10^12/L increase	−0.582	0.133	0.559	0.430–0.726	<0.001
BG	Per 1 mmol/L increase	0.073	0.032	1.076	1.010–1.146	0.024

Variable	Retained in original model	Selection frequency, %	Direction consistency, %
Panel C. Bootstrap variable-selection frequencies			
Onset-to-admission time	Yes	100.0	100.0
rSIG	Yes	100.0 (forced)	100.0
RBC	Yes	79.8	99.9
Cerebral herniation	Yes	78.7	100.0
BG	Yes	73.8	100.0
Lymphocytes	No	54.2	—
Cr	No	54.2	—
Temp	No	39.4	—
Ca	No	32.0	—
TC	No	28.7	—
Hb	No	26.5	—
LDL-C	No	25.3	—
INR	No	23.2	—
Alb	No	23.0	—
APTT	No	21.3	—
UA	No	17.9	—
TBil	No	16.9	—
HDL-C	No	16.9	—
AST	No	14.9	—
ALP	No	11.6	—

AIC, Akaike information criterion; APTT, activated partial thromboplastin time; Alb, albumin; ALP, alkaline phosphatase; aOR, adjusted odds ratio; AST, aspartate aminotransferase; AUC, area under the receiver operating characteristic curve; BG, blood glucose; Ca, calcium; CI, confidence interval; Cr, creatinine; GCS, Glasgow Coma Scale; Hb, hemoglobin; HDL-C, high-density lipoprotein cholesterol; HR, heart rate; INR, international normalized ratio; LDL-C, low-density lipoprotein cholesterol; OR, odds ratio; RBC, red blood cell count; rSIG, reverse shock index multiplied by Glasgow Coma Scale score; SBP, systolic blood pressure; SE, standard error; TBil, total bilirubin; TC, total cholesterol; Temp, body temperature; UA, uric acid.

When rSIG was categorized into quartiles, the association was concentrated primarily in the lowest quartile. Using Q4 (>27.6340) as the reference, patients in Q1 (≤17.6345) had markedly higher odds of HE in the unadjusted model (OR, 17.514; 95% CI, 8.785–34.913), the fully adjusted model (aORs, 8.841; 95% CI, 4.221–18.518), and the stepwise model (aORs, 8.407; 95% CI, 4.088–17.289). Q2 and Q3 were not significantly different from Q4. Trend tests remained significant in all prespecified and stepwise models (all P for trend <0.001), indicating that the inverse association was driven mainly by the substantially elevated risk among patients with the lowest rSIG values (Table 3).

**Table 3:** Association between rSIG quartiles and HE across prespecified and stepwise logistic regression models.

rSIG quartile	Model 1 OR (95% CI)	Model 2 aOR (95% CI)	Model 3 aOR (95% CI)	Model 4 aOR (95% CI)	Stepwise model aOR (95% CI)
Q1 (≤17.6345)	17.514 (8.785–34.913)	14.208 (7.017–28.767)	14.764 (7.263–30.011)	8.841 (4.221–18.518)	8.407 (4.088–17.289)
Q2 (>17.6345–23.2940)	1.684 (0.730–3.883)	1.648 (0.701–3.876)	1.688 (0.717–3.974)	1.479 (0.624–3.507)	1.471 (0.630–3.438)
Q3 (>23.2940–27.6340)	1.118 (0.450–2.774)	1.144 (0.457–2.863)	1.159 (0.463–2.901)	1.075 (0.427–2.707)	1.084 (0.432–2.722)
Q4 (>27.6340)	1.000 (Reference)	1.000 (Reference)	1.000 (Reference)	1.000 (Reference)	1.000 (Reference)
Trend OR per 5-point increase	0.382 (0.326–0.447)	0.416 (0.354–0.488)	0.412 (0.350–0.484)	0.489 (0.412–0.580)	0.499 (0.422–0.591)
P for trend	<0.001	<0.001	<0.001	<0.001	<0.001
P overall	<0.001	<0.001	<0.001	<0.001	<0.001

APTT, activated partial thromboplastin time; aOR, adjusted odds ratio; BG, blood glucose; CI, confidence interval; Hb, hemoglobin; INR, international normalized ratio; OR, odds ratio; PLT, platelet count; Q, quartile; RBC, red blood cell count; rSIG, reverse shock index multiplied by Glasgow Coma Scale score.

**Figure 5:**
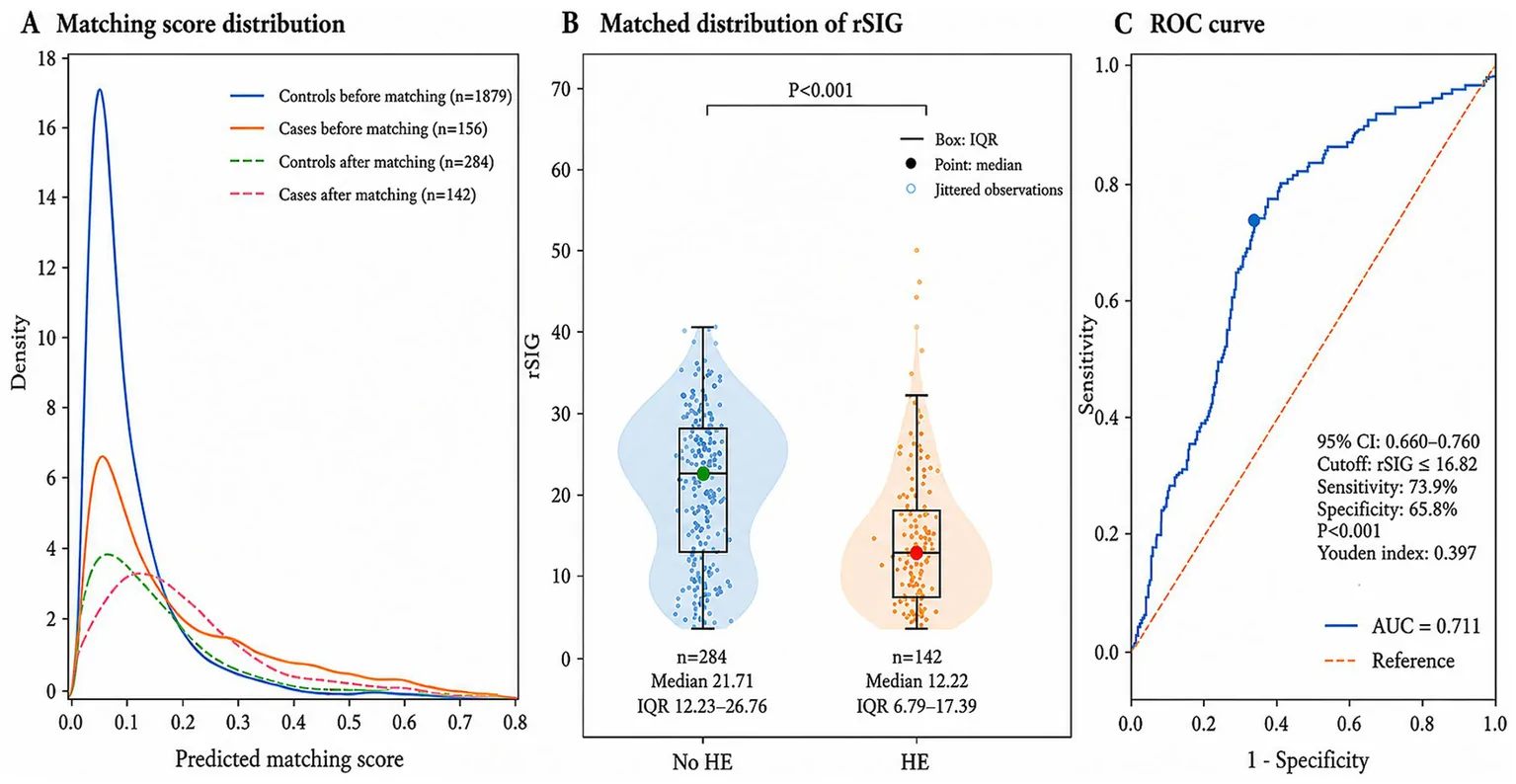
Matched sensitivity analysis and post-matching discriminatory performance of rSIG. **(A)** Distribution of matching scores before and after 1:2 matching. A total of 142 patients with HE were matched to 284 patients without HE. **(B)** Violin plot of rSIG in the matched cohort. The violin plot is overlaid with boxplots, median points, jittered observations, and sample sizes. The median rSIG was 21.71 (IQR, 12.23–26.76) in matched controls and 12.22 (IQR, 6.79–17.39) in matched patients with HE; the between-group difference was significant (*p* < 0.001, Mann–Whitney *U* test). **(C)** Receiver operating characteristic curve of the matched-cohort logistic regression model using rSIG as the sole predictor. The AUC was 0.711 (95% CI, 0.660–0.760; *p* < 0.001). The optimal cutoff was rSIG ≤ 16.82, with a sensitivity of 73.9% and a specificity of 65.8%.

In the 1:2 propensity score-matched sensitivity analysis, 142 of the 156 patients with HE were successfully matched to 284 patients without HE. As shown in Figure 5A, the distributions of propensity scores became substantially more comparable between the HE and non-HE groups after matching. Despite adjustment for these baseline differences, admission rSIG remained markedly lower in patients with HE than in matched controls [12.22 (IQR, 6.79–17.39) vs. 21.71 (IQR, 12.23–26.76), *p* < 0.001; Figure 5B]. In conditional logistic regression stratified by matched set, each 5-point decrease in rSIG was associated with higher odds of HE (conditional OR, 1.660; 95% CI, 1.425–1.933; p < 0.001). The direction and magnitude of this association were broadly consistent with those observed in the primary multivariable analyses, indicating that the association between lower admission rSIG and HE remained robust after propensity score matching. In the matched cohort, rSIG retained moderate discriminatory ability, with an AUC of 0.711 (95% CI, 0.660–0.760); the optimal cutoff was 16.82, yielding a sensitivity of 73.9% and a specificity of 65.8% (Figure 5C). These findings support the stability of rSIG as an exposure associated with HE after balancing measured baseline covariates, although the residual imbalance in age should be considered when interpreting the matched analysis.

## Discussion

In this single-center retrospective cohort of 2,035 patients with sICH, lower admission rSIG was consistently associated with a higher risk of early HE. This relationship remained evident after adjustment for demographic characteristics, onset-to-admission time, comorbidities, laboratory variables, and indicators of disease severity. The association was nonlinear and was most pronounced among patients with low rSIG values, suggesting that the index may be particularly useful for identifying a high-risk subgroup. The rSIG-only model also showed good discrimination, acceptable calibration, and a measurable clinical net benefit. Similar results were obtained in the hierarchical models, quartile analysis, bootstrap resampling, and matched sensitivity analysis. Taken together, these findings support rSIG as a simple bedside marker that may complement early clinical and imaging assessment in patients with sICH.

These findings accord with previous evidence that rSIG reflects the combined effects of neurological impairment and hemodynamic instability. Park et al. reported a nonlinear association between lower rSIG and in-hospital mortality in 318,506 trauma patients, with a cutoff of 16.5 (19). Kokeguchi et al. later validated a cutoff of 16.21 in isolated severe traumatic brain injury for urgent intervention, critical care, and mortality (20). Other trauma studies also supported rSIG as a rapid triage tool, although it was not consistently superior to more complex scores (21). Similar prognostic value has been reported in suspected sepsis, where lower rSIG identified patients requiring early resuscitation and predicted short-term outcomes (22, 23), and in ICU patients with heatstroke (24). Unlike these studies, this study work examined early hematoma expansion rather than mortality or treatment intensity. It therefore extends rSIG to non-traumatic sICH, although the similarity in reported cutoffs does not establish a universal threshold.

The biological basis of this association probably lies in what the components of rSIG capture rather than in a direct effect of the index. A low GCS may reflect greater brain injury, mass effect, rising intracranial pressure, and disruption of the central autonomic network. This disturbance can produce sympathetic predominance, tachycardia, blood pressure fluctuation, and stress hyperglycemia after ICH (25). Greater HR variability and hyperacute SBP variability have both been associated with HE (26, 27). Rapid hemodynamic oscillations may increase transmural stress on recently ruptured vessels and interfere with early clot stabilization. Stress hyperglycemia may further promote oxidative stress, endothelial injury, and coagulation disturbance; a higher stress hyperglycemia ratio has been independently associated with early HE (28). At the molecular level, blood products activate glial cells, increase inflammatory cytokines and reactive oxygen species, and induce matrix metalloproteinases, leading to extracellular-matrix degradation and blood–brain barrier disruption. Early MMP-9 elevation has been linked to HE (29), while human studies have demonstrated neuroinflammation and blood–brain barrier breakdown after acute ICH (30). Thus, low rSIG may mark the convergence of neurological injury, autonomic dysregulation, vascular instability, and inflammatory barrier damage, although these mechanisms remain inferential.

The nonlinear pattern in our cohort adds practical meaning to the association. HE risk increased sharply at the lower end of rSIG and was driven mainly by the lowest quartile, whereas the curve flattened at higher values. This suggests that rSIG may be more useful as a warning marker for a high-risk subgroup than as a uniformly graded predictor. A similar concentration of risk in selected score ranges has been reported for the BAT score and other HE prediction scores (31, 32). The rSIG-only model showed good discrimination, acceptable calibration, and clinical net benefit using only GCS, SBP, and HR. Although its AUC of 0.828 was numerically within the range reported for several clinical–imaging scores, nomograms, and radiomics models, such cross-study comparisons are indirect because of differences in patient populations, imaging intervals, definitions of HE, and validation methods (9, 10, 33–35). The principal potential contribution of rSIG therefore lies in its speed, parsimony, and accessibility rather than in proven superiority of discrimination. CTA and NCCT markers directly characterize active bleeding or hematoma morphology, clinical–imaging scores integrate several radiological and clinical predictors, and radiomics models extract high-dimensional imaging features (36). By contrast, rSIG reflects the immediate neurological and hemodynamic state using only three bedside measurements. It can also be readily recalculated, although the present study evaluated only the first admission value. This operational simplicity may be useful during the earliest phase of emergency assessment, while detailed imaging interpretation is pending, or in settings where CTA or radiomics infrastructure is unavailable. Nevertheless, the research did not perform head-to-head comparisons, net reclassification analyses, or formal model-updating analyses to determine whether rSIG adds predictive information beyond established HE scores. Accordingly, rSIG should be viewed as a complementary screening marker rather than a substitute for imaging-based assessment. Prospective multicenter studies should directly compare rSIG with established prediction tools and determine whether its incorporation improves discrimination, calibration, and clinical decision-making.

Given these findings, rSIG may be most useful as an admission screening tool rather than a stand-alone decision rule. Patients with low values could be prioritized for closer neurological observation, timely repeat CT, and review of blood pressure, glucose, temperature, and anticoagulant exposure. This is clinically relevant because the INTERACT3 care bundle improved outcomes through early protocolized management (37). Hyperacute hemostatic trials, TICH-2 and TRAIGE, also relied on treatment within 8 h and rapid identification of patients at risk of HE (38, 39). In factor Xa inhibitor-associated intracerebral hemorrhage, ANNEXA-I showed that rapid reversal reduced hematoma expansion, although thrombotic events increased (40). Study strengths include a large consecutive cohort, first admission measurements, standardized follow-up NCCT within 8 h, blinded review by two neurosurgeons, and consistent findings across prespecified, nonlinear, bootstrap, and matched analyses. These features support rSIG as a low-cost adjunct to, rather than a replacement for, clinical and imaging assessment.

## Limitations

Several limitations should be acknowledged. First, the retrospective, single-center design may have introduced selection bias and residual confounding despite multivariable adjustment, and the findings may not be fully generalizable to other populations or care settings. Second, early HE occurred in a relatively small proportion of patients, which may have reduced the precision of subgroup and matched analyses. Third, rSIG was calculated from a single set of admission measurements and may have been influenced by prehospital treatment, sedation, intubation, pain, agitation, or transient hemodynamic changes. Fourth, several established imaging predictors, including hematoma location and morphology, intraventricular extension, and the CTA spot sign, were not fully incorporated into the models. Detailed medication-related variables, including antiplatelet therapy, anticoagulant reversal, and hemostatic treatment, were also unavailable or incompletely recorded. Fifth, hematoma volume was estimated using the ABC/2 method, which may be less accurate for irregularly shaped hemorrhages. Future studies will incorporate software-assisted three-dimensional hematoma segmentation and radiomics-based imaging analysis to achieve more precise volumetric and morphological assessment. Sixth, the study relied on internal validation and matched sensitivity analysis without an independent external cohort. Therefore, the proposed cutoff should be considered exploratory and requires prospective multicenter validation before routine clinical application. Moreover, rSIG was not directly compared with established clinical or imaging-based HE prediction tools, and its incremental predictive value beyond existing models remains uncertain. Finally, because rSIG incorporates GCS, SBP, and HR, we did not determine whether the composite index provides greater predictive value than these individual components alone. Future multicenter prospective studies should externally validate rSIG, directly compare it with its individual components and established HE prediction tools, and determine whether incorporating rSIG into imaging- or laboratory-based models meaningfully improves prediction and clinical decision-making. Given the observational nature of the study, the association between lower rSIG and HE should not be interpreted as causal.

## Conclusion

Lower admission rSIG was independently associated with a higher risk of early hematoma expansion in patients with spontaneous intracerebral hemorrhage. The association was nonlinear and most pronounced at low rSIG values. As a simple index derived from routinely available admission measurements, rSIG may support rapid bedside risk stratification and complement imaging assessment. However, its cutoff and incremental clinical value require confirmation in prospective multicenter cohorts before routine clinical application.

## Data Availability

The datasets presented in this article are not readily available because they contain potentially identifiable clinical information and are subject to institutional and ethical restrictions. De-identified data may be made available from the corresponding author upon reasonable request and following approval by the Ethics Committee of the Second Affiliated Hospital of Fujian Medical University. Requests to access the datasets should be directed to JW: wjy_ysq@163.com.

## Supplementary material

Supplementary Figure S1 SHAP interpretation of the final backward stepwise multivariable LR model.
