# Supplementary material for: Lower admission reverse shock index multiplied by Glasgow coma scale score is associated with early hematoma expansion in spontaneous intracerebral hemorrhage
Source: Front Neurol. 2026 Sep 1;17:1947152. doi: 10.3389/fneur.2026.1947152 (PMC13574643; doi:10.3389/fneur.2026.1947152)
Supplement: Supplementary file 1 [file Table_1.docx]

**Supplementary Table S1 Baseline characteristics according to HE status**

| **Variable** | **Overall (N=2,035)** | **Non-HE (n=1,879)** | **HE (n=156)** | **P value** |
| --- | --- | --- | --- | --- |
| Age, years | 57 (45–68) | 57 (45–69) | 57 (46–67) | 0.987 |
| Male sex, n (%) | 1370 (67.3) | 1264 (67.3) | 106 (67.9) | 0.862 |
| Smoking, n (%) | 208 (10.2) | 195 (10.4) | 13 (8.3) | 0.418 |
| Alcohol use, n (%) | 163 (8.0) | 155 (8.2) | 8 (5.1) | 0.168 |
| Onset-to-admission time | 13 (6–72) | 12 (6–48) | 96 (10–2160) | <0.001 |
| GCS, points | 15.0 (12.0–15.0) | 15.0 (13.0–15.0) | 7.5 (4.0–10.2) | <0.001 |
| BT, °C | 36.6 (36.5–36.9) | 36.6 (36.5–36.8) | 36.8 (36.5–37.0) | <0.001 |
| HR, beats/min | 80.0 (72.0–88.0) | 80.0 (72.0–88.0) | 84.5 (78.0–97.0) | <0.001 |
| SBP, mmHg | 134.0 (121.0–150.0) | 134.0 (121.0–150.0) | 131.5 (120.0–146.2) | 0.243 |
| Hypertension, n (%) | 516 (25.4) | 482 (25.7) | 34 (21.8) | 0.287 |
| Diabetes mellitus, n (%) | 301 (14.8) | 282 (15.0) | 19 (12.2) | 0.339 |
| Cerebral herniation, n (%) | 119 (5.8) | 88 (4.7) | 31 (19.9) | <0.001 |
| PLT, ×10⁹/L | 198.0 (158.5–243.0) | 199.0 (161.0–243.0) | 183.0 (134.0–244.2) | 0.011 |
| Hb, g/L | 126.0 (111.0–139.0) | 127.0 (112.0–140.0) | 104.5 (92.8–124.2) | <0.001 |
| RBC, ×10¹²/L | 4.12 (3.66–4.57) | 4.16 (3.70–4.59) | 3.41 (2.97–4.22) | <0.001 |
| ALC, ×10⁹/L | 1.244 (0.884–1.711) | 1.265 (0.899–1.726) | 0.972 (0.742–1.384) | <0.001 |
| HDL-C, mmol/L | 1.13 (0.90–1.41) | 1.15 (0.91–1.42) | 0.91 (0.77–1.29) | <0.001 |
| LDL-C, mmol/L | 2.53 (1.97–3.16) | 2.56 (2.00–3.17) | 2.16 (1.75–2.93) | <0.001 |
| Cr, μmol/L | 65.0 (53.0–78.3) | 66.0 (54.0–78.3) | 55.8 (35.8–78.5) | <0.001 |
| UA, μmol/L | 282.0 (217.0–360.0) | 286.0 (219.0–361.0) | 249.5 (181.8–348.2) | 0.002 |
| ALP, U/L | 70.0 (57.0–87.0) | 70.0 (57.0–86.0) | 78.0 (60.8–106.2) | <0.001 |
| Ca, mmol/L | 2.18 (2.10–2.26) | 2.18 (2.10–2.26) | 2.17 (2.04–2.25) | 0.007 |
| TBil, μmol/L | 12.40 (8.70–17.02) | 12.60 (8.90–17.23) | 9.71 (6.09–14.32) | <0.001 |
| AST, U/L | 23.0 (17.0–34.1) | 23.0 (17.0–33.0) | 26.0 (17.0–40.3) | 0.027 |
| Alb, g/L | 39.6 (36.5–42.7) | 39.9 (36.8–42.7) | 36.2 (32.7–40.6) | <0.001 |
| BG, mmol/L | 6.01 (5.17–7.61) | 5.97 (5.16–7.52) | 6.53 (5.29–8.62) | 0.007 |
| TC, mmol/L | 4.10 (3.49–4.80) | 4.14 (3.54–4.81) | 3.81 (3.21–4.61) | <0.001 |
| INR | 1.02 (0.96–1.09) | 1.01 (0.96–1.09) | 1.05 (1.00–1.13) | <0.001 |
| APTT, s | 27.7 (25.6–32.3) | 27.6 (25.6–32.2) | 29.9 (26.5–34.3) | 0.002 |
| rSIG | 23.294 (17.634–27.634) | 23.731 (18.854–27.924) | 11.382 (6.333–16.664) | <0.001 |

**Abbreviations:** ALC, absolute lymphocyte count; ALP, alkaline phosphatase; Alb, albumin; APTT, activated partial thromboplastin time; AST, aspartate aminotransferase; BG, blood glucose; BT, body temperature; Ca, calcium; Cr, creatinine; GCS, Glasgow Coma Scale; Hb, hemoglobin; HDL-C, high-density lipoprotein cholesterol; HR, heart rate; INR, international normalized ratio; IQR, interquartile range; LDL-C, low-density lipoprotein cholesterol; PLT, platelet count; Q1, first quartile; Q3, third quartile; RBC, red blood cell count; rSIG, reverse shock index multiplied by the Glasgow Coma Scale score; SBP, systolic blood pressure; SD, standard deviation; TBil, total bilirubin; TC, total cholesterol; UA, uric acid.
