# Supplementary material for: Lower admission reverse shock index multiplied by Glasgow coma scale score is associated with early hematoma expansion in spontaneous intracerebral hemorrhage
Source: Front Neurol. 2026 Sep 1;17:1947152. doi: 10.3389/fneur.2026.1947152 (PMC13574643; doi:10.3389/fneur.2026.1947152)
Supplement: Supplementary file 2 [file Table_2.docx]

**Supplementary Table S2 Shapiro-Wilk normality tests for continuous variables by HE status**

| **Variable** | **Non-HE W** | **P-value P value** | **HE W** | **P-value** |
| --- | --- | --- | --- | --- |
| Age, years | 0.989 | <0.001 | 0.983 | 0.048 |
| Onset-to-admission time | 0.305 | <0.001 | 0.666 | <0.001 |
| GCS, points | 0.594 | <0.001 | 0.884 | <0.001 |
| BT, °C | 0.847 | <0.001 | 0.863 | <0.001 |
| HR, beats/min | 0.958 | <0.001 | 0.961 | <0.001 |
| SBP, mmHg | 0.981 | <0.001 | 0.944 | <0.001 |
| PLT, ×10⁹/L | 0.957 | <0.001 | 0.882 | <0.001 |
| Hb, g/L | 0.986 | <0.001 | 0.979 | 0.019 |
| RBC, ×10¹²/L | 0.993 | <0.001 | 0.975 | 0.007 |
| ALC, ×10⁹/L | 0.956 | <0.001 | 0.890 | <0.001 |
| HDL-C, mmol/L | 0.978 | <0.001 | 0.911 | <0.001 |
| LDL-C, mmol/L | 0.990 | <0.001 | 0.961 | <0.001 |
| Cr, μmol/L | 0.888 | <0.001 | 0.945 | <0.001 |
| UA, μmol/L | 0.980 | <0.001 | 0.949 | <0.001 |
| ALP, U/L | 0.844 | <0.001 | 0.922 | <0.001 |
| Ca, mmol/L | 0.990 | <0.001 | 0.978 | 0.013 |
| TBil, μmol/L | 0.914 | <0.001 | 0.823 | <0.001 |
| AST, U/L | 0.639 | <0.001 | 0.780 | <0.001 |
| Alb, g/L | 0.986 | <0.001 | 0.984 | 0.076 |
| BG, mmol/L | 0.805 | <0.001 | 0.855 | <0.001 |
| TC, mmol/L | 0.991 | <0.001 | 0.968 | 0.001 |
| INR | 0.867 | <0.001 | 0.847 | <0.001 |
| APTT, s | 0.909 | <0.001 | 0.942 | <0.001 |
| rSIG | 0.985 | <0.001 | 0.880 | <0.001 |

**Abbreviations:** ALC, absolute lymphocyte count; ALP, alkaline phosphatase; Alb, albumin; APTT, activated partial thromboplastin time; AST, aspartate aminotransferase; BG, blood glucose; BT, body temperature; Ca, calcium; Cr, creatinine; GCS, Glasgow Coma Scale; Hb, hemoglobin; HDL-C, high-density lipoprotein cholesterol; HR, heart rate; INR, international normalized ratio; IQR, interquartile range; LDL-C, low-density lipoprotein cholesterol; PLT, platelet count; Q1, first quartile; Q3, third quartile; RBC, red blood cell count; rSIG, reverse shock index multiplied by the Glasgow Coma Scale score; SBP, systolic blood pressure; SD, standard deviation; TBil, total bilirubin; TC, total cholesterol; UA, uric acid.
