# Supplementary material for: Lower admission reverse shock index multiplied by Glasgow coma scale score is associated with early hematoma expansion in spontaneous intracerebral hemorrhage
Source: Front Neurol. 2026 Sep 1;17:1947152. doi: 10.3389/fneur.2026.1947152 (PMC13574643; doi:10.3389/fneur.2026.1947152)
Supplement: Supplementary file 3 [file Table_3.docx]

**Supplementary Table S3 Univariable and final stepwise multivariable LR analyses of factors associated with HE**

| **Variable** | **Effect scale** | **Univariable OR (95% CI)** | ***P* value** | **Final stepwise model aOR (95% CI)** | ***P* value** |
| --- | --- | --- | --- | --- | --- |
| Age | Per 10-year increase | 0.998 (0.905–1.100) | 0.964 | — | — |
| Sex | Male vs female | 1.031 (0.727–1.463) | 0.862 | — | — |
| Onset-to-admission time | Per 100-minute increase | 1.064 (1.052–1.076) | <0.001 | 1.053 (1.039–1.067) | <0.001 |
| Smoking | Yes vs no | 0.785 (0.437–1.412) | 0.419 | — | — |
| Alcohol use | Yes vs no | 0.601 (0.290–1.248) | 0.172 | — | — |
| Body temperature | Per 1°C increase | 1.906 (1.469–2.473) | <0.001 | — | — |
| Hypertension | Yes vs no | 0.808 (0.545–1.198) | 0.288 | — | — |
| Diabetes mellitus | Yes vs no | 0.785 (0.478–1.290) | 0.340 | — | — |
| Cerebral herniation | Yes vs no | 5.047 (3.226–7.898) | <0.001 | 1.893 (1.098–3.262) | 0.022 |
| PLT | Per 50 × 10⁹/L increase | 0.991 (0.888–1.107) | 0.878 | — | — |
| Hb | Per 10 g/L increase | 0.696 (0.644–0.751) | <0.001 | — | — |
| RBC count | Per 1 × 10¹²/L increase | 0.313 (0.247–0.395) | <0.001 | 0.559 (0.430–0.726) | <0.001 |
| ALC | Per 1 × 10⁹/L increase | 0.544 (0.404–0.732) | <0.001 | — | — |
| HDL-C | Per 1 mmol/L increase | 0.238 (0.141–0.400) | <0.001 | — | — |
| LDL-C | Per 1 mmol/L increase | 0.656 (0.534–0.806) | <0.001 | — | — |
| Creatinine | Per 10 μmol/L increase | 0.824 (0.757–0.897) | <0.001 | — | — |
| Uric acid | Per 100 μmol/L increase | 0.818 (0.698–0.958) | 0.013 | — | — |
| ALP | Per 10 U/L increase | 1.109 (1.060–1.160) | <0.001 | — | — |
| Calcium | Per 0.1 mmol/L increase | 0.807 (0.718–0.907) | <0.001 | — | — |
| Total bilirubin | Per 10 μmol/L increase | 0.581 (0.439–0.768) | <0.001 | — | — |
| AST | Per 10 U/L increase | 1.065 (1.008–1.125) | 0.024 | — | — |
| Albumin | Per 5 g/L increase | 0.563 (0.484–0.656) | <0.001 | — | — |
| Blood glucose | Per 1 mmol/L increase | 1.085 (1.028–1.144) | 0.003 | 1.076 (1.010–1.146) | 0.024 |
| Total cholesterol | Per 1 mmol/L increase | 0.710 (0.595–0.848) | <0.001 | — | — |
| INR | Per 0.1 increase | 1.307 (1.174–1.455) | <0.001 | — | — |
| APTT | Per 5-second increase | 1.259 (1.094–1.449) | 0.001 | — | — |
| rSIG | Per 5-point decrease | 2.212 (1.970–2.483) | <0.001 | 1.746 (1.534–1.988) | <0.001 |
| AUC | — | — | — | 0.864 | — |
| Brier score | — | — | — | 0.053 | — |
| AIC | — | — | — | 797.1 | — |

**Abbreviations:** AIC, Akaike information criterion; ALC, absolute lymphocyte count; ALP, alkaline phosphatase; APTT, activated partial thromboplastin time; AST, aspartate aminotransferase; AUC, area under the receiver operating characteristic curve; BG, blood glucose; CI, confidence interval; GCS, Glasgow Coma Scale; Hb, hemoglobin; HDL-C, high-density lipoprotein cholesterol; HE, hematoma expansion; INR, international normalized ratio; LDL-C, low-density lipoprotein cholesterol; OR, odds ratio; aOR, adjusted odds ratio; PLT, platelet count; RBC, red blood cell count; rSIG, reverse shock index multiplied by Glasgow Coma Scale score; SBP, systolic blood pressure.
